# 

**Published:** 1874-11

**Authors:** 


### Books and Pamphlets Received.

Cyclopædia of the Practice of Medicine. Edited by Dr. H. von Ziemssen. Vol. I. Acute Infectious Diseases, by Prof. Liebermeister, Prof. Lebert, Dr. Heuisch, Prof Heubner and Dr. Oertel. The American Translation; edited by Albert H. Buck, M. D. New York: Wm. Wood & Co., 1874.

Transactions of the American Medical Association for 1874.

Clinical Lectures on Diseases on the Urinary Organs; By Sir Henry Thompson. Philadelphia: Henry C. Lea, 1874. Buffalo: T. Butler & Son.

A Guide to the Practical Examination of the Urine; By James Tyson, M.D Philadelphia: Lindsay & Blakiston.

The Chemists' and Druggists' Diary for 1874. London: Office of the Chemist and Druggist.

The Longevity of Brain-workers; By Geo. M. Beard, M. D. New York.

The treatment of Marasums, Whooping Cough and Debility of Children by Electricity; By Geo. M. Beard, M. D. From Detroit Review of Med. and Pharmacy, October, 1874.

Cases of Hysteria, Neurasthenia, Spinal Irritation, and allied Affections; with remarks; By Geo. M. Beard, M. D. From Chicago Journal of Nervous and Mental Diseases.

### Medical and Surgical Journal

PUBLISHED MONTHLY,

Containing Original Articles, Reports of Medical Societies and Hospitals, Editorial, Reviews, Correspondence, Army News, etc., etc ; including the usual variety of Medical Periodical Publications. Specimen copies sent on application.

Address Buffalo Medical & Surgical Journal, Buffalo, N. Y.

TERMS OF SUBSCRIPTION, - - $3.00 PER YEAR, IN ADVANCE.

